# Purinergic Signaling in Pathologic Osteogenic Differentiation of Aortic Valve Interstitial Cells from Patients with Aortic Valve Calcification

**DOI:** 10.3390/biomedicines11020307

**Published:** 2023-01-21

**Authors:** Polina Klauzen, Daria Semenova, Daria Kostina, Vladimir Uspenskiy, Anna Malashicheva

**Affiliations:** 1 Laboratory of Regenerative Biomedicine, Institute of Cytology Russian Academy of Science, Tikhoretskiy Avenue, 4, Saint Petersburg 194064, Russia; 2 Almazov National Medical Research Centre, Akkuratova Street, 2, Saint-Petersburg 197341, Russia

**Keywords:** aortic valve, calcification, purinergic signaling, osteogenic differentiation

## Abstract

Purinergic signaling is associated with a vast spectrum of physiological processes, including cardiovascular system function and, in particular, its pathological calcifications, such as aortic valve stenosis. Aortic valve stenosis (AS) is a degenerative disease for which there is no cure other than surgical replacement of the affected valve. Purinergic signaling is known to be involved in the pathologic osteogenic differentiation of valve interstitial cells (VIC) into osteoblast-like cells, which underlies the pathogenesis of AS. ATP, its metabolites and related nucleotides also act as signaling molecules in normal osteogenic differentiation, which is observed in pro-osteoblasts and leads to bone tissue development. We show that stenotic and non-stenotic valve interstitial cells significantly differ from each other, especially under osteogenic stimuli. In osteogenic conditions, the expression of the ecto-nucleotidases ENTPD1 and ENPP1, as well as ADORA2b, is increased in AS VICs compared to normal VICs. In addition, AS VICs after osteogenic stimulation look more similar to osteoblasts than non-stenotic VICs in terms of purinergic signaling, which suggests the stronger osteogenic differentiation potential of AS VICs. Thus, purinergic signaling is impaired in stenotic aortic valves and might be used as a potential target in the search for an anti-calcification therapy.

## 1. Introduction

Calcific aortic valve stenosis (AS) is a degenerative disease for which there is no cure other than surgical replacement of the affected valve. Pathological conditions associated with the disease include mechanical stress, endothelial dysfunction, lipid deposition, oxidative stress, bicuspid aortic valve, inflammation, extracellular matrix remodeling and biomineralization (comprehensively reviewed in [1]). Purinergic signaling is associated with a vast spectrum of physiological processes, including cardiovascular system function and also with its pathological calcification, such as calcific aortic valve stenosis.

It is known that the pathologic osteogenic differentiation of valve interstitial cells (VIC) into osteoblast-like cells underlies the pathogenesis of AS, and purinergic signaling is involved in this [2]. During normal osteogenic differentiation, which is observed in pro-osteoblasts and leads to bone tissue development, ATP, its metabolites and related nucleotides also act as signaling molecules.

Purinergic signaling involves ATP, its metabolites and related molecules, which may be implicated in multidirectional cellular effects. For example, while ATP and ADP act as damage-associated molecular patterns (DAMPs) and induce sterile inflammatory response, adenosine and inosine are known to be anti-inflammatory agents. Regarding vascular and valvular calcification, it has been shown that ATP and UTP decrease osteogenic the differentiation of VICs and vascular smooth muscles cells (VSMC), while uridine adenosine tetraphosphate (Up4A) stimulates it [3].

Purin metabolites are formed during their degradation by ecto-nucleotidases, which produce derivative products also exhibiting their effects via purinergic receptors. The first ectonucleotidase in the chain of extracellular ATP metabolites is ENTPD1 (CD39, E-NTPDase1), which converts ATP to AMP and phosphate (Pi). AMP is further converted to adenosine by the ectonucleotidase NT5E (CD73, Ecto5’NTase) with a release of inorganic phosphate (Pi); after that, adenosine is converted to inosine by the ectonucleotidase ADA. ENTPD1 and NT5E are significantly expressed in two main types of valve cells, namely, valve endothelial cells (VEC) and VICs. Cell cultivation with an addition of extracellular nucleotides showed a more rapid formation of NT5E products on VICs than on VECs, while the opposite pattern was found for ENTPD1 [4].

There is also an alternative mechanism of nucleotide degradation in which ATP and some other purines may be degraded to AMP by another ectonucleotidase—ENPP1 (CD203a)—with a release of pyrophosphate (PPi). While Pi promotes hydroxyapatite deposition, Ppi is known for its inhibiting influence on hydroxyapatite deposition via chemisorption of pyrophosphate on the surface of hydroxyapatite, which prevents further crystal growth. Nevertheless, PPi may be converted to Pi by alkaline phosphatase. ATP may decrease the calcification rate via PPi production. In addition, ATP decreases calcification via reduction in apoptosis [3], which is known to trigger vascular calcification by serving apoptotic bodies as nucleating structures for calcium crystal formation [5]. It has been shown that ENPP1 is highly upregulated in AS VICs and, despite the production of PPi, promotes the mineralization of cells [3,6]. This might be due to the fact that the overexpression of ENPP1 depletes the pool of extracellular ATP, which acts as a survival signal for VICs and prevents apoptosis [3]. In this way, apoptosis, which triggers calcium crystal formation, might be a stronger factor of calcification than Pi/PPi production.

Nucleotides act in cells via specific receptors. P2 purinergic receptors (P2X and P2Y subtypes) are mainly sensitive to ATP and UTP, while P1 (ADORA) receptors are activated by adenosine and inosine. The precise role of the purinergic receptors in cardiovascular calcification is controversial and not clear. Several works show an inhibitory effect of P2Y2 receptors, activated by ATP, on vascular calcification through PI3K/Akt [3,7]. In contrast, other works showed that the effects of ATP and UTP on VSMC calcification were not mediated via the P2Y2 receptor [8]. In addition, an activation of P2Y2/6 receptors by Up4A was described, which involved phosphorylation of the mitogen-activated kinases MEK and ERK1/2, followed by enhanced calcification of vascular cells [9,10].

Adenosine and its receptor ADORA2b may play a major role in cardiovascular calcification. Adenosine is a mediator of intercellular signaling in many tissues. In the cardiovascular system, adenosine reduces heart rate, inhibits inflammation, protects cells from hypoxia caused by this inflammation and increases resistance to ischemia [11]. Adenosine also impacts AS pathogenesis. It has been reported that stimulating receptors ADORA2A and ADORA2B produces strong pro-degenerative effects on VICs [12]. There are data showing that the expression of ADORA2a and ADORA2b is diminished in uncalcified fragments of stenotic valves compared to non-stenotic ones [13]. It has also been shown that the activity of adenosine deaminase, which degrades adenosine, is increased in stenotic aortic valves [13]. This leads to increased vascular adenosine degradation and hypertension. Due to turbulent blood flow on the aortic side of the valve, its stenotic part is prone to attachment and infiltration of immune cells with subsequent endothelial damage and even more severe calcification.

VICs play a crucial role in valvular calcification and are thought to differentiate into osteoblast-like cells. Nevertheless, the process is orchestrated by other cell types, such as VECs. Multiple effects of endothelial cells on the underlying interstitial cells have been shown. Endothelial cells biosynthesize Up4A via the activation of vascular endothelial growth factor receptor (VEGFR). Up4A synthesis performed by the endothelium is enhanced by stimulation with ATP, UTP and mechanical stress [14], which takes place in aortic stenosis. Up4A has a much longer half-life compared to ATP/UTP, contains both purine and pyrimidine parts [14] and, in contrast to ATP/UTP, enhances vascular calcification in rat VSMCs [9].

This study is devoted to the identification of the distinctive features of cells derived from stenotic aortic valves in the context of purinergic signaling, as well as searching for similarities and differences between the processes of pathogenic and normal osteogenic differentiation of mesenchymal cells. We show here that AS valvular cells react to extracellular ATP and adenosine in a distinct manner in comparison to non-stenotic cells; AS VICs also undergo pathogenic osteogenic differentiation more similar to osteoblast-like cells compared to the osteogenic differentiation of non-stenotic cells. Elevated expression levels of ATP-degrading nucleotidases and adenosine receptors indicate a general elevation of purinergic signaling in AS VICs influenced and orchestrated by the endothelial cells. Nevertheless, some components of the signaling turn out to be downregulated. Our data indicate a general imbalance in the purinergic signaling in AS VICs.

## 2. Materials and Methods

### 2.1. Isolation of Primary Cells

The study protocols were approved by the local Ethics Committee of Almazov Federal Medical Research Centre and Vreden Institute of Traumatology and Orthopedics and performed in accordance with the principles of the Declaration of Helsinki. All patients gave written informed consent. Human valve interstitial cells (VIC) and human valve endothelial cells (VEC) were isolated from tricuspid aortic valves explanted during aortic valve replacement due to calcific aortic valve disease (CAVD). Patients with known infective endocarditis and rheumatic disease were excluded from the study. Clinical data of the patients are represented in Table 1. VICs and VECs from normal aortic valves, which were used as a control group, were isolated from healthy tricuspid aortic valves obtained from explanted hearts from recipients of heart transplantation. Human umbilical vein endothelial cells (HUVEC) were obtained at Almazov National Medical Research Center from cords after healthy delivery. Femur bone epiphysis for osteoblast-like cell isolation was acquired during surgery at Vreden Institute of Traumatology and Orthopedics [15].

VICs and VECs were isolated with a standard technique. Leaflets of the heart valves were cleared from aorta fragments. VECs were isolated by vortexing collagenase−treated leaflets (10 min with collagenase type IV) (Worthington Biochemical Corporation, Lakewood, NJ, USA). The resulting pellet after vortexing was suspended and seeded in a flask covered with 0.2% gelatin in an ECM (ScienCell, Logan, UT, USA). When a VEC confluence of 70–80% was attained, cells were magnetically sorted by CD31 antigen and passaged in a ratio of 1:3 in the ECM (ScienCell, Logan, UT, USA). To isolate VICs, leaflets were incubated again in collagenase type IV overnight. The resulting pellet after vortexing was suspended and seeded in a flask in DMEM supplemented with 15% FBS, 2 mM of L-glutamine and penicillin/streptomycin (100 mg/L) (all—Invitrogen, Grand Island, NJ, USA).

HUVEC were acquired from the umbilical vein by collagenase dissociation. The vein was filled with 0.1% collagenase solution (Collagenase, Type II) (Worthington Biochemical Corporation, USA) and incubated in PBS at 37 °C for 10 min. The suspension was centrifuged at 300× *g* for 5 min. The cell pellet was suspended and seeded on a Petri dish covered with 0.2% gelatin in the ECM (ScienCell, Logan, UT, USA).

Isolation of osteoblast-like cells from bone tissue was carried out using the enzymatic method. Bone tissue was crushed into fragments using carbide cutters. Cancellous bone fragments were selected, washed in PBS and incubated with 0.2% collagenase type II (Worthington Biochemical Corporation, USA) solution for 30 min at 37 °C. The homogenized mass was washed in PBS and transferred into 0.2% collagenase type IV solution (Worthington Biochemical Corporation, USA) for 16 h at 37 °C. The collagenase type IV was then inactivated with high-glucose DMEM (Gibco, Grand Island, NJ, USA) supplemented with 15% FBS (HyClone, New York, NY, USA) and 0.1% ascorbic acid solution (Sigma-Aldrich, St. Louis, MO, USA). The resulting suspension was transferred into a flask and cultivated for several weeks. Then, the resulting osteoblast-like cells were cultured in Petri dishes according to standard procedures [15].

### 2.2. Culture and Osteogenic Differentiation

For the experiments, VICs, VECs and HUVECs of 3–5 passages were used. The cells were seeded in 6-well plates with a density of 220 × 103 cells per well. Osteoblasts of 3–4 passages were plated in 12-well plates with a density of 90 × 103 cells per well covered with 0.2% gelatin. VICs and VICs with HUVECs were cultured under normal conditions in DMEM supplemented with 15% bovine serum (all—Gibco, USA), 2 mM glutamine, 50 IU/mL penicillin and 50 IU/mL streptomycin (Invitrogen, USA). VEC cultivation was carried out using an ECM medium (ScienCell, USA) with the addition of the appropriate supplements from the manufacturer. Osteoblasts were cultured in 4.5% glucose DMEM with the addition of 15% Hyclone bovine serum (all—Gibco, USA), 2 mM glutamine, 50 IU/mL penicillin and 50 IU/mL streptomycin (Sigma, USA). To induce osteogenic differentiation, cells were placed in DMEM medium supplemented with 10% Hyclone bovine serum (Gibco, USA), 2 mM glutamine, 50 IU/mL penicillin, 50 IU/mL streptomycin, 100 mM b-glycerophosphate, 50 mg/mL ascorbic acid and 0.1 µM dexamethasone (Sigma, USA).

All cells were cultured for 4 days, after which total RNA was isolated using the ExtractRNA reagent (Evrogen, Moscow, Russia) in accordance with the manufacturer’s recommendations. Calcium deposition was detected by Alizarin Red staining (Sigma, USA) on day 18 of differentiation for the VIC and on day 15 for osteoblast cells.

### 2.3. Real-Time PCR

Real-time PCR was used to assess the expression levels of exo-nucleotidase and purinergic receptors. PCRs for the VEC and VIC populations (pure and in co-cultivation with HUVEC) were run without technical and biological replicates. PCRs were performed in technical and biological duplicates; all the resulting values were independently used in the analysis. Total RNA (1 μg) was reverse-transcribed with an MMLV RT kit (Eurogen, Moscow, Russia). Real-time PCR was performed with 1 μL of cDNA and TaqMan PCR Mastermix (Thermo Fisher Scientific, Grand Island, NJ, USA) in the Light Cycler system. The thermocycling conditions were as follows: 95 °C for 5 min, followed by 45 cycles at 95 °C for 15 s and 60 °C for 1 min. A final heating step of 65 °C to 95 °C was performed to obtain melting curves of the final PCR products. The corresponding gene expression level was normalized to GAPDH from the same samples. Changes in the target genes expression levels were calculated as fold differences using the comparative ΔΔCT method. ENTPD1, ENPP1, ADA, NT5E, ADORA2b, P2RX1 and P2RX7 primers were bought at Thermofisher Scientific (USA) as Single Tube TaqMan Gene Expression Assays (Hs01054040_m1, Hs00159686_m1, Hs00969559_m1, Hs01110945_m1, Hs00386497_m1, Hs00175721_m1, Hs00175686_m1, Hs00925146_m1, respectively).

Values were compared using Student’s *t*-test and nonparametric Spearman correlation. A value of *p* ≤ 0.05 was considered significant. Statistical analysis was performed using R software (version 2.12.0; R Foundation for Statistical Computing, Vienna, Austria).

## 3. Results

### 3.1. Purinergic Signaling in Osteogenic Differentiation of Healthy and Stenotic Aortic Valve Interstitial Cells Versus Osteoblasts

**Figure 1:**
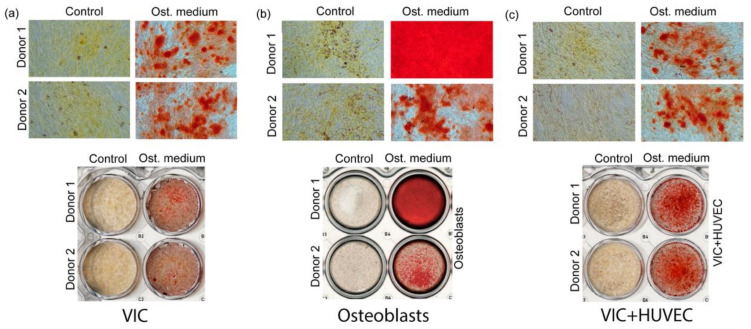
Microscopic images of alizarin-red staining of cells after osteogenic differentiation; calcium depositions are stained in red. Valve interstitial cells (VIC) (**a**), osteoblasts (**b**) and VIC co-cultivated with human umbilical cord endothelial cells (HUVEC) (**c**) before and after osteogenic differentiation.

As pathologic osteogenic differentiation of VICs underlies the pathogenesis of AS, we induced the osteogenic differentiation of VICs to simulate this process. In all differentiated cell lineages, calcium depositions were positively stained (Figure 1).

**Figure 2:**
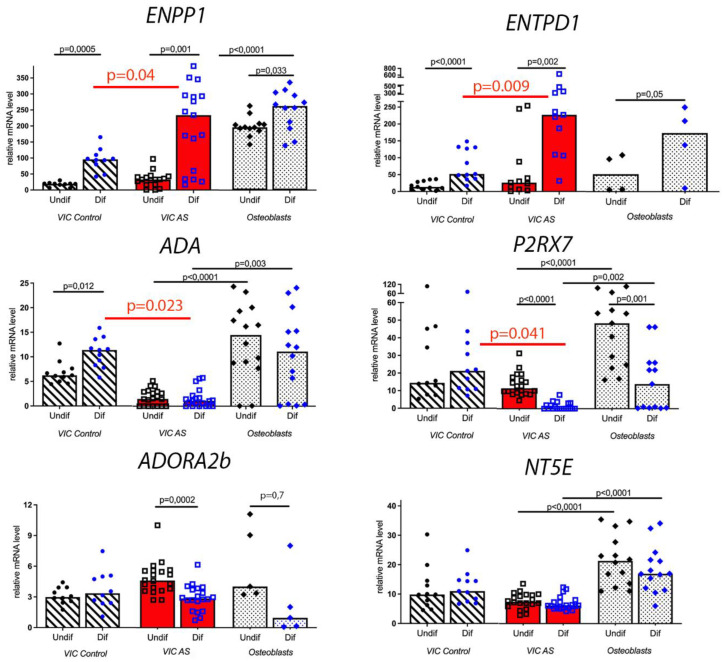
Expression levels of ENPP1, ENTPD1, ADA, P2RX7, ADORA2b and NT5E in valve interstitial cells (VIC) and osteoblast-like cells. Relative mRNA levels in non-stenotic VICs (VIC Control) are compared with stenotic VICs (AS VIC); relative mRNA levels in stenotic VICs are compared with osteoblast-like cells (Osteoblasts). Relative mRNA levels in undifferentiated cells (Undif) are compared with cells in which osteogenic differentiation (Dif) was induced.

Osteogenic conditions strongly increased the expression of the ecto-nucleotidases ENPP1 and ENTPD1 in both AS and non-stenotic VICs (Figure 2). Importantly, this elevation in ENPP1 and ENTPD1 expression in osteogenic media was higher in VICs derived from AS patients compared to the control group. Osteogenic differentiation also raised the expression of the ecto-nucleotidase ADA in non-stenotic cells and reduced the expression level of the ATP receptor P2RX7 and the adenosine receptor ADORA2b in AS VICs.

Osteoblast-like cells normally undergo osteogenic differentiation. We analyzed the expression profile of the components of purinergic signaling in human osteoblasts in osteogenic conditions (Figure 2). In differentiated osteoblasts, we observed an elevated expression level of the ecto-nucleotidases ENPP1 and ENTPD1 similarly to AS and non-stenotic VICs. The ADA level in osteoblast-like cells was not changed. The expression level of the ATP receptor P2RX7 declined in differentiated osteoblasts similarly to AS VICs. Concurrently, the expression level of adenosine receptor ADORA2b remained the same in osteogenic conditions and in non-stenotic cells, but not in AS VIC. The osteogenic differentiation of osteoblasts did not influence the expression of the ecto-nucleotidase NT5E, similarly to that in VICs.

To summarize, pro-osteoblast differentiation looks similar to VIC differentiation in terms of purinergic signaling component expression and in most cases corresponds to AS VICs but not to non-stenotic control cells. Nevertheless, the expression levels of ENPP1, ADA, NT5E and P2RX7 in osteoblast-like cells are reliably higher than in VICs, both before and after osteogenic differentiation. At the same time, the expression level of ADORA2b is not elevated in osteoblasts compared to VICs.

Osteogenic conditions help to explain the difference between AS and non-stenotic cells regarding the expression levels of purinergic signaling components. In our experiments (Figure 2), the osteogenic medium triggered elevated expression of the ecto-nucleotidases ENTPD1 and ENPP1 in AS VICs compared to non-stenotic cells and a decreased expression of the ecto-nucleotidase ADA and ATP receptor P2RX7.

### 3.2. Purinergic Signaling in Valve Endothelial Cells

**Figure 3:**
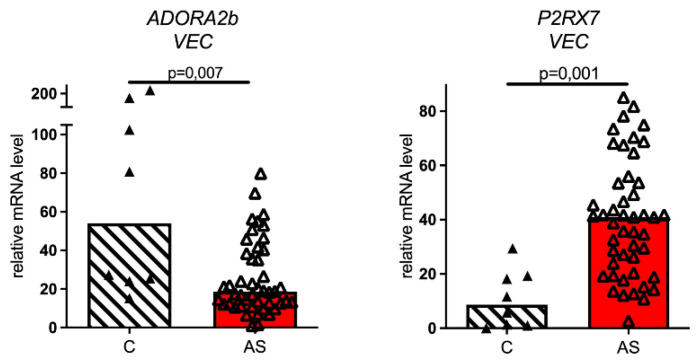
Expression levels of ADORA2b and P2RX7 in VEC. Relative mRNA levels in non-stenotic control cells (C) are compared with relative mRNA levels in stenotic cells (AS).

We also analyzed the expression of purinergic genes in valve endothelial cells (VEC) (Figure 3). In VEC, the expression level of ADORA2b also varied between AS and non-stenotic cells, but, in contrast to VIC, its expression decreased in AS cells compared to the control cells from healthy valves. In addition, AS VEC displayed an elevated level of the ATP receptor P2RX7 compared to non-stenotic VEC.

**Figure 4:**
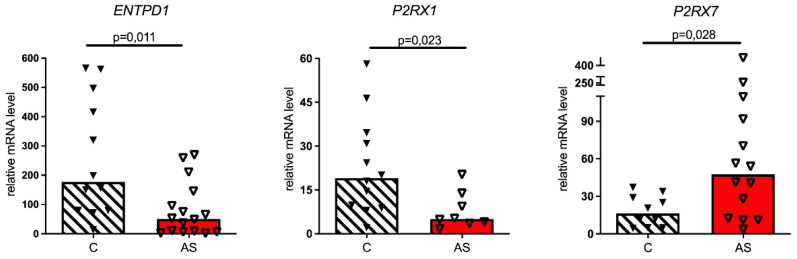
Expression levels of ENTPD1, P2RX1 and P2RX7 in VIC co-cultured with endothelial cells. Relative mRNA levels in non-stenotic control cells (C) are compared with relative mRNA levels in stenotic cells (AS).

To see whether the presence of endothelial cells could influence purinergic signaling in VICs, we analyzed the expression of purinergic genes in a co-culture of VICs and endothelial cells. A co-culture of VICs with HUVECs revealed the difference between AS and non-stenotic VICs (Figure 4). The expression levels of the ecto-nucleotidase ENTPD1 and ATP-receptor P2RX1 in AS were lower compared to non-stenotic cells in the co-culture. Notably, this was not observed in a pure VIC culture (Figure 2). At the same time, the expression of ATP-receptor P2RX7 was higher in stenotic VICs co-cultured with endothelial cells in comparison to control cells from healthy valves (Figure 4).

The small molecule adenosine is a purine nucleoside widely used as a vasodilator and neuromodulator. Recently, a growing amount of attention has been focused on adenosine and adenosine receptors as factors implicated in bone regeneration. Exogenous adenosine supplementation has been reported as an effective method for successful osteogenic differentiation of mouse bone-marrow-derived MSC [16]. Therefore, we analyzed whether exogenous adenosine influenced osteogenic differentiation of valve interstitial cells. We induced the osteogenic differentiation of VICs in the presence of 10 uM of adenosine and analyzed whether that influenced alizarin staining efficiency. We did not detect any influence of adenosine on the effectivity of osteogenic differentiation of VICs. However, we observed a change in the expression profile of CD39/CD73 (ENTPD1/NT5E) using flow cytometric analysis (Supplementary Figure S1).

In summary, our data suggest an important role that changes in purinergic signaling could play in the sensitivity of cells from diseased valves undergoing pathologic osteogenic differentiation.

## 4. Discussion

**Figure 5:**
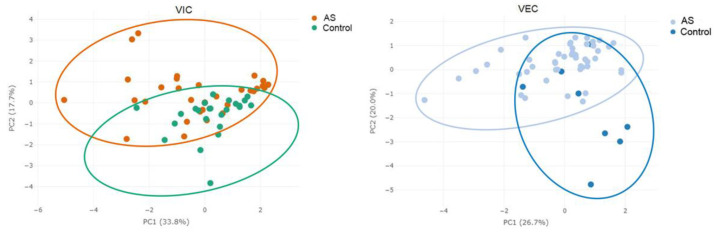
Principal component analysis (PCA) plot representing ENTPD1, ENPP1, ADA, NT5E, P2RX1, P2RX7 and ADORA2b gene expression in non-stenotic (Control) and stenotic (AS) VICs and VECs.

We found that aortic valve cells derived from stenotic patients significantly differ in purinergic signaling from their counterparts derived from healthy donors. This fact is also proven by PCA plotting, which shows that different kinds of cells (AS and non-stenotic) create two clusters (Figure 5). Valve interstitial cells from stenotic and non-stenotic valves significantly differ in their expression of ENTPD1, ADORA2b and P2RX7 genes, while endothelial cells from stenotic and non-stenotic valves mainly differ in their expression of genes ADORA2b, P2RX7 and P2RX1.

The endothelial influence on purinergic signaling component expression in VICs and their pathological differentiation potential is significant. It is known that the ecto-nucleotidase ENTPD1 is involved in the catabolism of the dinucleotide Up4A [17], which is produced by the endothelium and increases the intensity of the pathological calcification process in the cardiovascular system [9].

In the course of ATP degradation, the ectonucleotidases ENTPD1 and NT5E release Pi, while ENPP1 releases PPi—an inhibitor of hydroxyapatite formation and, hence, a mineralization deprivator. The relative amounts of Pi and PPi play an important role in the development of tissue calcification. In a co-culture of VICs with endothelial cells, the ENTPD1-pathway also predominates because endothelium increases ENTPD1 but does not affect ENPP1. It turns out that the endothelium changes the balance of two ATP-degradation pathways in VIC and hence reduces the relative amount of pyrophosphate and increases the relative amount of phosphate. Thus, the endothelium of the diseased valves could induce hydroxyapatite deposits.

It has been reported that the P2RX7 receptor participates in the differentiation of osteoclasts and osteoblasts and its downregulation inhibits osteogenic potential of pre-osteoblastic cells [18]. It also serves as a pattern recognition receptor for extracellular ATP-mediated apoptotic cell death [19]. Our results evidence a significant increase in P2RX7 expression in normal conditions in AS VECs and AS VICs co-cultured with endothelium (Figure 3 and Figure 4). Nevertheless, pure AS VIC in normal conditions do not display this over-expression, nor do AS cells and osteoblasts in the osteogenic medium where we observed the downregulation of P2RX7. These data may indicate that in osteogenic conditions, when the work of ENTPD1 and ENPP1 is strongly intensified in AS VICs, the pool of extracellular ATP is largely exhausted and the signal does not reach the P2RX7 receptor. Here, our data suggest that in normal conditions AS VECs, unlike AS VICs intensify P2RX7 expression. Since the P2RX7 receptor takes part in ATP-mediated apoptotic cell death, AS VECs may intensify the apoptosis-mediated mineralization of VICs. These data further indicate the crucial orchestrating role of endothelium in the pathological calcification of VICs.

Adenosine, an important signaling molecule and a stress regulator, is produced by the ecto-nucleotidase NT5E from AMP. It is known that increasing adenosine levels produces strong pro-degenerative effects in VIC, and vice versa, lowering adenosine levels by the inhibition of NT5E leads to protective effects against VIC osteogenic degeneration [12].

According to our results, NT5E expression in VICs, VECs and pro-osteoblasts remained constant in all experiments, even in osteogenic conditions when AMP production was reinforced (elevated ENTPD1 and ENPP1 in osteo-differentiated cells, Figure 2). However, some evidence suggests that NT5E expression varies in non-stenotic and AS valves tissues when analyzed using immunohistochemical or immunofluorescent staining [13]. Changing NT5E expression might represent an effect of different cell type interactions in valve tissue and could be influenced by other tissue components, such as inflammatory cells, while isolated and co-cultured VICs and VECs exhibit stable NT5E expression. In addition, there are data showing that cells isolated from different locations of stenotic valves express different levels of NT5E [13], although in our work, we did not take the latter parameter into consideration.

We show that ecto-nucleotidase ADA, which is supposed to degrade over-accumulated adenosine, is not elevated in AS VICs in normal conditions, as might be expected. Moreover, its expression is even decreased in AS VICs under osteogenic stimulation (Figure 2). It is known that adenosine might be deaminated by adenosine deaminase, whose activity in AS cells is higher than in non-stenotic cells [20]. So, while normal VICs tend to reduce extra adenosine level via ADA, AS cells probably reduce extra adenosine with the help of adenosine deaminase.

Adenosine takes part in cardiovascular pathologies through the activation of adenosine receptors, including ADORA2b. It has been reported that stimulating adenosine receptors ADORA2a and ADORA2b results in strong pro-osteogenic effects on VICs [12]. We show that ADORA2b expression was elevated in AS VICs, both in normal and osteogenic conditions (Figure 2). The intensified ADORA2b pathway in AS VICs suggests their strong potential for osteogenic transition and also the elevated amount of adenosine in the stenotic valve, which is an important stress marker. Despite the stable NT5E expression observed in our experiments, adenosine level may be increased in stress caused by stenosis.

This study has limitations associated with the 2D in vitro culture of isolated valve cells and also with the fact that we isolated the cells from a patient with end-stage disease. Unfortunately, the animal models for calcific aortic valve stenosis are still very limited [21], and this lack of relevant models limits our understanding of the disease pathogenesis. Nevertheless, we suggest that our findings are relevant for finding potential targets for calcific aortic stenosis, as understanding the mechanisms of pathological osteogenic differentiation of valve cells could help to find potential anti-calcification therapies [22,23].

## 5. Conclusions

We show here that valve interstitial cells from stenotic valves react to extracellular ATP and adenosine in a distinct manner in comparison to non-stenotic valve cells. AS VICs also undergo pathologic osteogenic differentiation more similarly to osteoblast-like cells in comparison to cells from non-stenotic valves. Elevated expression levels of ATP-degrading nucleotidases and adenosine receptors suggest general elevation of purinergic signaling in AS VICs, influenced and orchestrated by the endothelial cells. However, some components of the signaling were found to be downregulated. Our data indicate a general imbalance in the purinergic signaling in aortic valve interstitial cells of the stenotic valves. Moreover, in osteogenic conditions, the diseased interstitial cells look more similar to osteoblasts than non-stenotic cells in terms of purinergic signaling, which suggest the stronger osteogenic differentiation potential of AS VICs. In summary, purinergic signaling is impaired in stenotic aortic valves and might be used as a potential target in the search for anti-calcification therapies.

## Figures and Tables

**Table 1:** Clinical data of the patients.

Characteristics (m, w)	CAVD Patients (n = 17) (m, w)
Age	63.0 (58.0; 67.0)
Gender	W—15 (46.8%) M—17 (53.2%)
Proximal ascending aortic diameter, mm	36 (32; 40)
Body mass index (BMI) kg/m^2^	32.62 (26.79; 34.94)
General cholesterol, mM/L	4.91 (4.23; 6.11)
High density lipoproteins (HDL), mM/L	1.16 (1.04; 1.46)
Low density lipoproteins (LDL), mM/L	3.54 (2.77; 4.17)
C-reactive protein, mg/L	3.01 (1.06; 4.70)

## Data Availability

All data related to this work can be made available upon request to the corresponding authors.

## Supplementary Materials

The following supporting information can be downloaded at: https://www.mdpi.com/article/10.3390/biomedicines11020307/s1. Figure S1: Analysis of the influence of adenosine on the expression of CD39 (ENTPD1) and CD73 (NT5E) in valve interstitial cells from the patients with aortic stenosis. The cells were induced to osteogenic differentiation in control conditions and in the presence of 10 uM of adenosine. CD39/CD73 expression was analyzed by flow cytometry using specific antibodies to CD39 and CD73 (Biolegend) and flowcytometer Cytoflex. * *p* < 0.01, ** *p* < 0.05.
