# Supplementary material for: Purinergic Signaling in Pathologic Osteogenic Differentiation of Aortic Valve Interstitial Cells from Patients with Aortic Valve Calcification
Source: Biomedicines. 2023 Jan 21;11(2):307. doi: 10.3390/biomedicines11020307 (PMC9953532; doi:10.3390/biomedicines11020307)
Supplement: Supplementary file 1 [file biomedicines-11-00307-s001.zip › biomedicines-2137713-supplementary.pdf]

Supplement

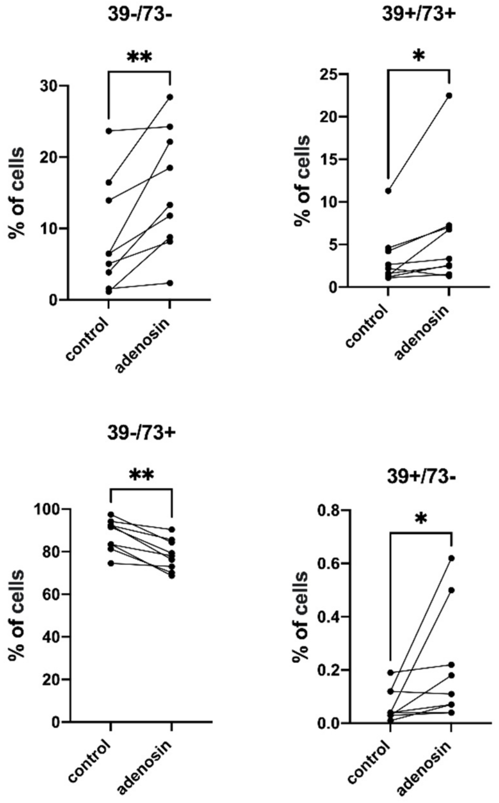

Supplementary figure 1
Analysis of the influence of adenosine on the expression of CD39 (*ENTPD1*) and CD73 (*NT5E*) in valve interstitial cells from the patients with aortic stenosis. The cells were induced to osteogenic differentiation in control conditions and in the presence of 10 uM of adenosine. CD39/CD73 expression was analyzed by flow cytometry using specific antibodies to CD39 and CD73 (Biolegend) and flowcytometer Cytoflex. *p<0,01, **p<0,05
